# Growth on Alpha-Ketoglutarate Increases Oxidative Stress Resistance in the Yeast* Saccharomyces cerevisiae*

**DOI:** 10.1155/2017/5792192

**Published:** 2017-01-05

**Authors:** Maria Bayliak, Nadia Burdyliuk, Volodymyr Lushchak

**Affiliations:** Department of Biochemistry and Biotechnology, Vasyl Stefanyk Precarpathian National University, 57 Shevchenko Str., Ivano-Frankivsk 76018, Ukraine

## Abstract

Alpha-ketoglutarate (AKG) is an important intermediate in cell metabolism, linking anabolic and catabolic processes. The effect of exogenous AKG on stress resistance in* S. cerevisiae* cells was studied. The growth on AKG increased resistance of yeast cells to stresses, but the effects depended on AKG concentration and type of stressor. Wild-type yeast cells grown on AKG were more resistant to hydrogen peroxide, menadione, and transition metal ions (Fe^2+^ and Cu^2+^) but not to ethanol and heat stress as compared with control ones. Deficiency in SODs or catalases abolished stress-protective effects of AKG. AKG-supplemented growth led to higher values of total metabolic activity, level of low-molecular mass thiols, and activities of catalase and glutathione reductase in wild-type cells compared with the control. The results suggest that exogenous AKG may enhance cell metabolism leading to induction of mild oxidative stress. It turn, it results in activation of antioxidant system that increases resistance of* S. cerevisiae* cells to H_2_O_2_ and other stresses. The presence of genes encoding SODs or catalases is required for the expression of protective effects of AKG.

## 1. Introduction

In all aerobic organisms, reactive oxygen species (ROS) are continuously generated as side products of cellular metabolism. These species, namely, superoxide anion radical (O_2_^•−^), hydrogen peroxide (H_2_O_2_), and hydroxyl radical (HO^•^), are potentially dangerous due to their high reactivity and capability to interact virtually with all cellular components including proteins, lipids, nucleic acids, and carbohydrates. For defense against ROS, cells contain antioxidant enzymes such as superoxide dismutase (SOD), catalase, and several peroxidases, as well as thiol-containing antioxidants like glutathione and thioredoxin. Under some circumstances, the balance between ROS generation and their elimination is disturbed leading to enhanced ROS level called “oxidative stress”. This imbalance can result from exposure to diverse environmental stress conditions such as the presence of oxidants, heat shock, ethanol, and metal ions. Oxidative stress development plays a pivotal role in aging and numerous diseases, including cancer and cardiovascular and neurodegenerative diseases [1, 2]. Therefore, there has been a growing interest in low-molecular mass natural antioxidant molecules that prevent and alleviate the effects of oxidative stress [3–5].

Many studies report on the antioxidant properties of alpha-ketoglutarate (AKG) which is an important intermediate in the Krebs cycle. Primarily, protective effects of AKG were established in vitro systems. In particular, AKG prevented H_2_O_2_-induced hemolysis of human erythrocytes [6] and iron-induced lipid peroxidation in rat brain in vitro [7]. An antioxidant action of AKG was proposed to be mediated through nonenzymatic interaction of AKG with H_2_O_2_ with formation of succinate, carbon dioxide, and water [6, 8]. Subsequently, the antioxidant mode of AKG was found to be involved in its protective effects against different toxicants. According to this, dietary AKG prevented the increase in the levels of oxidative stress markers in rats under chronic ethanol administration [9] and cyanide treatment [10] and in* Drosophila melanogaster* flies exposed to sodium nitroprusside [11].

In addition to direct antioxidant function, recent studies show that AKG increased the capacity of endogenous antioxidant system [12, 13]. Experiments with human erythrocytes demonstrated that AKG could serve as glutamate source for the synthesis of glutathione, an important low-molecular mass thiol antioxidant [14]. This ketoacid also stabilized redox homeostasis in aged mice via stimulation of antioxidant defense [12]. Moreover, AKG supplement was found to increase lifespan in nematode* Caenorhabditis elegans* [15] and this effect was accompanied by concomitant increase in ROS generation. The prooxidant effect of AKG seems to be due to the altered intensity of the Krebs cycle in which AKG is preferentially metabolized [16, 17]. A small increase in ROS level may be beneficial for an organism due to the induction of mild oxidative stress which results in stimulation of defense systems with increasing adaptive capacity [1, 18–20]. In line with this, the activation of antioxidant defense and the enhanced cold tolerance were found in* D. melanogaster* flies grown on AKG-supplemented food [13].

Previously we showed that AKG increased resistance of budding yeast* S. cerevisiae* to hydrogen peroxide in combined treatment [8]. In this work, we aimed at studying an adaptive potential of* S. cerevisiae* cells grown in the medium supplemented with AKG. For this, control and AKG-grown cells from exponentially growing cultures were transferred to a new medium followed by treatment with different stressors, and yeast survival was monitored. We investigated also if the protective effects of AKG were associated with the induction of endogenous antioxidant defense. Yeast mutants defective in certain components of antioxidant system were used to reveal the key endogenous antioxidants required for realization of the effects of AKG.

## 2. Materials and Methods

### 2.1. Chemicals

Phenylmethylsulfonyl fluoride (PMSF), ethylenediaminetetraacetic acid (EDTA), 2,4-dinitrophenylhydrazine (DNPH), 5,5′-dithiobis-2-nitrobenzoic acid (DTNB), 2,3,5-triphenyl tetrazolium chloride (TTC), oxidized glutathione (GSSG), NADPH, FeSO_4_  ×  7H_2_O, and menadione were obtained from Sigma-Aldrich Corporation (USA); diagnostic kits for determination of glucose were from Cormay (Łomianki, Poland); N,N,N′,N′-tetramethyl ethylenediamine (TEMED) and quercetin were from Reanal (Hungary); peptone and yeast extract were from Fluka (Germany). All other chemicals were of analytical grade.

### 2.2. Strains and Growth Conditions

The yeast strains used in this study were the wild-type strain YPH250 (*MAT ***a*** trp1-*Δ*1 his3-*Δ*200 lys2-801 leu2-*Δ*1 ade2-101 ura3-52*) and its isogenic mutants Δ*sod1* (as YPH250 but* sod1::URA3*), Δ*sod1*Δ*sod2* (as YPH250 but* sod1::kanMX2 sod2*Δ*::TRP1*),* Δcta1Δctt1* (as YPH250 but* cta1::TRP1 ctt1::URA3*), Δ*yap1* (as YPH250 but* yap1-*Δ*1::HIS3*), and Δ*gsh1* (as YPH250 but* gsh1::LEU2*). All strains were kindly provided by Dr. Yoshiharu Inoue (Kyoto University, Japan). Cells were grown at 28°C with shaking at 175 rpm in liquid medium YPD containing 1% (w/v) yeast extract, 2% (w/v) peptone, 2% (w/v) glucose, and different concentrations (mM) of sodium salt of *α*-ketoglutarate (AKG). pH value of the medium was adjusted to 5.5. The initial concentration of cells in the medium was about 0.3 × 10^6^ cells/mL. Cell growth was measured as an increase in optical density at 620 nm (OD_620_) with microplate reader Multiskan MCC/340 (Labsystems, Helsinki, Finland). The concentration of glucose in the medium free from cells was determined using Liquick Cor-glucose kit. Results were expressed as percentage (w/v) of glucose in the medium.

### 2.3. Stress Treatment

Exponentially growing yeast cells were harvested after 18 h of cultivation, washing, and resuspension in equal volume of 50 mM potassium phosphate buffer (pH 7.0) without or with adding 10 mM AKG. Then, the aliquots of experimental suspensions were exposed to different stressors for 30 min at 28°C. Yeast cells were exposed to the following stressors: 2 mM FeSO_4_, 2 mM CuSO_4_, 20% C_2_H_5_OH, 100 mM menadione, and heat shock (40°C). Control cells were incubated under the same conditions but without stressors. Cell survival after stress exposure was evaluated by counting the number of colony-forming units on YPD agar plates.

### 2.4. Preparation of Cell-Free Extracts, Assay of Antioxidant Enzyme Activities, Protein Carbonyls, Low-Molecular Mass Thiols, and Total Protein

After 18 h of growth, cells from experimental or control cultures were collected by centrifugation at room temperature (5 min, 5000*g*) and washed with 50 mM potassium phosphate buffer (pH 7.0). The yeast pellets were resuspended in lysis buffer (50 mM potassium phosphate buffer, 1 mM phenylmethylsulfonyl chloride, and 0.5 mM EDTA). Cell extracts were prepared by vortexing yeast suspensions with glass beads (0.5 mm) as described earlier [21] and kept on ice for immediate use.

The measurement of activities of superoxide dismutase (SOD), catalase, and glutathione reductase (GR) was conducted as described earlier [21]. The parameters were measured with a Spekol 211 spectrophotometer (Carl Zeiss, Germany) and SF-46 (LOMO, USSR). The activity of catalase was measured by monitoring disappearance of hydrogen peroxide at 240 nm using the extinction coefficient for hydrogen peroxide of 39.4 M^−1^ cm^−1^ [21]. The activity of catalase was assayed in 2 mL of medium containing 50 mM potassium phosphate buffer (pH 7.0), 0.5 mM EDTA, 10 mM H_2_O_2_, and 3 *µ*L of supernatant obtained as described above. The activity of SOD was assayed at 406 nm as the inhibition of quercetin oxidation by superoxide anion in the medium containing (final concentrations) 30 mM Tris-HCl buffer (pH 9.0), 0.5 mM EDTA, 0.8 mM TEMED, 50 *µ*M quercetin, and 1–25 *µ*L of cell extract in a final volume of 1.5 mL [21]. One unit of SOD activity was defined as the amount of soluble protein of supernatant that inhibited the maximal rate of quercetin oxidation by 50%. The activity of glutathione reductase (GR) was measured following the consumption of NADPH in reaction medium containing 50 mM potassium phosphate buffer (pH 7.0), 0.5 mM EDTA, 1.0 mM oxidized glutathione, 0.25 mM NADPH, and 50 *µ*L of supernatant in a final volume of 1 mL. NADPH oxidation by GR was registered at 340 nm and the extinction coefficient for the coenzyme of 6220 M^−1^·cm^−1^ was used [21]. One unit of catalase and GR activities was defined as the amount of the enzyme consuming 1 *μ*mol of substrate per minute; the activities were expressed as international units (or milliunits) per milligram of the soluble protein.

The content of carbonyl groups in proteins (CP) was measured by determining the amount of 2,4-dinitrophenylhydrazone formed upon the reaction with 2,4-dinitrophenylhydrazine [22]. Carbonyl content was calculated from the absorbance maximum of 2,4-dinitrophenylhydrazone measured at 370 nm using an extinction coefficient of 22 × 10^3^ M^−1^·cm^−1^. The values are expressed as nanomoles of CP per milligram of protein.

Thiol-containing compounds were measured in cellular extracts by the light absorption of thiol conjugates with 5,5′-dithiobis-2-nitrobenzoic acid (DTNB) at 412 nm [23]. For determination of nonprotein low-molecular mass thiols (L-SH), aliquots of the cell extracts were mixed with 10% trichloroacetic acid (final concentration) and centrifuged (13,000*g*, 5 min, 21°C) to remove pelleted protein and the final supernatants were used for assay. For that, aliquots of the supernatant were incubated for 30 min with 50 *µ*M DTNB in 100 mM Tris-HCl buffer (pH 9.0). Absorption was read at 412 nm and a molar extinction coefficient of 14  ×  10^3^ M^−1^ cm^−1^ was used to calculate thiol level. Thiol levels were expressed as nanomoles of SH-groups per 10^8^ cells.

Protein concentration was determined by the Bradford assay with Coomassie G-250 [24] with bovine serum albumin as a standard.

### 2.5. Assay of Metabolic Activity

Yeast suspensions, containing 3  ×  10^8^ cells, were harvested by centrifugation at 3000*g* for 5 min and washed twice with distilled water with previous procedure. The yeast pellets were resuspended in 1 mL of 50 mM potassium phosphate buffer (pH 7.0) and mixed with 0.35 mL of 0.5% 2,3,5-triphenyl tetrazolium chloride (TTC). Metabolically active cells are capable of reducing the dye to a water-insoluble red formazan that was extracted from the cells with ethanol/acetone mixture (2 : 1), and the absorbance of this solution was measured at 485 nm [25, 26]. Metabolic activity was expressed in units of optical density at 485 nm per 10^8^ cells.

### 2.6. Native Polyacrylamide Gel Electrophoresis

Native polyacrylamide gel electrophoresis (PAGE) was performed on 3.6% stacking and 7.5% separating gels in standard Tris-glycine buffer (pH 8.3) according to the method of Davis [27]. The supernatants obtained as described above were mixed with glycerol (2 : 1) and the samples containing 10 *µ*g amount of total protein were applied to each well. Electrophoresis was performed at 200 V through the stacking gel for 30 min and 180 V through the separating gel for 5 h. Catalase activity was visualized by incubating the gels in 0.003% H_2_O_2_ for 15 min at room temperature, followed by treatment with solution, containing 2% (w/v) FeCl_3_ and 2% (w/v) [K_3_Fe(CN)_6_] [28]. Relative band intensities were estimated with TotalLab Quant Software.

### 2.7. Statistical Analysis

Experimental data are expressed as the mean value of 4–8 independent experiments ± the standard error of the mean (SEM). For statistical analysis of data, Student's* t*-test and Dunnett's test were used to compare samples with AKG versus control ones.

## 3. Results

### 3.1. Alpha-Ketoglutarate Does Not Affect Yeast Growth

**Figure 1:**
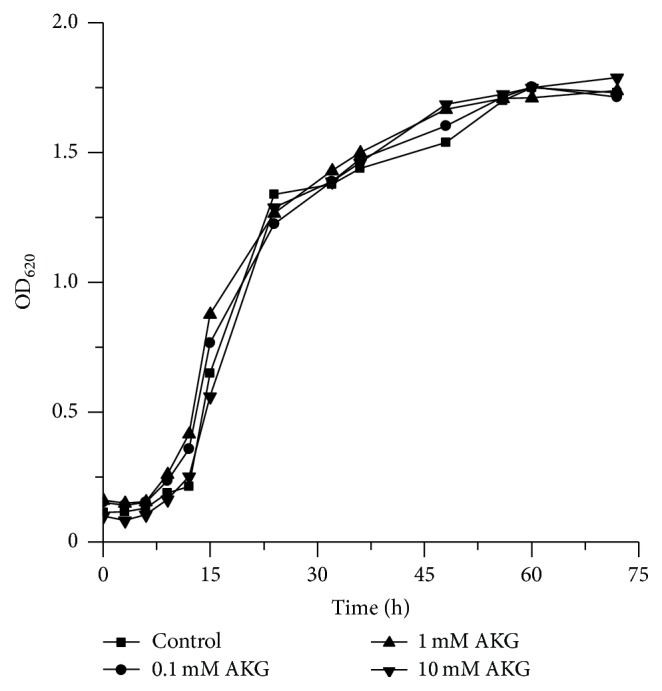
Growth curves of* S. cerevisiae* YPH250 in YPD medium in the presence of AKG at different concentrations. Growth was monitored by measuring the absorbance at 620 nm (OD_620_).

Many chemicals can affect yeast growth in dose-dependent manner being toxic at their higher levels [21, 29]. Therefore, we examined the yeast growth in YPD medium supplemented with different concentrations of AKG. The ketoacid at concentrations of 0.1–10.0 mM did not influence the growth rate of* S. cerevisiae* YPH250 (wild type) (Figure 1). Virtually, the same results were observed for mutant strains deficient in cytosolic and peroxisomal catalases (Δ*cta1*Δ*ctt1* strain), cytosolic Cu,Zn-SOD (Δ*sod1* strain), cytosolic Cu,Zn-SOD and mitochondrial Mn-SOD (Δ*sod1*Δ*sod2* strain), *γ*-glutamylcysteine synthetase, a key enzyme of glutathione biosynthesis (Δ*gsh1* strain), and oxidative stress regulator protein Yap1 (Δ*yap1* strain) (Table 1). The presence of AKG in the medium had little or no effect on growth rate of yeast mutants in batch cultures, although some differences were observed in growth rates between strains. The Δ*sod1* and Δ*sod1*Δ*sod2* strains grew more slowly than other strains, which is consistent with previous literature data [30].

### 3.2. Growth with AKG Increases Stress Resistance of Yeast Cells in Stressor- and Strain-Dependent Manner

**Figure 2:**
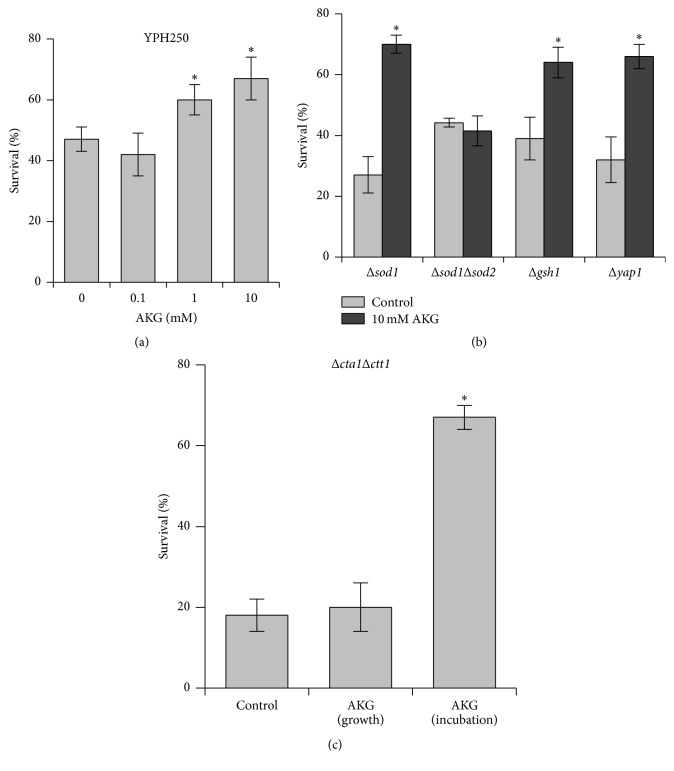
Effect of exposure to 10 mM H_2_O_2_ for 30 min on cell survival in* S. cerevisiae *YPH250 strain and its isogenic derivatives grown in the absence or presence of 10 mM AKG. (a) YPH250 cells (wild type) grown with AKG different concentrations; (b) mutant cells grown without or with 10 mM AKG; (c) Δ*cta1*Δ*ctt1* cells grown without or with AKG and were treated with only 10 mM H_2_O_2_ denoted as “control” and “AKG (growth),” respectively; “AKG (incubation),” Δ*cta1*Δ*ctt1* cells grown without AKG were treated with 10 mM H_2_O_2_ in combination with 10 mM AKG. Data are means ± SEM, *n* = 5-6. ^*∗*^Significantly different from respective control values with *P* < 0.05 using Dunnett's test (a) or Student's* t*-test (b, c).

Yeast cells grown without or with AKG were harvested at middle exponential phase, resuspended in 50 mM potassium phosphate buffer (pH 7.0), and then treated with different stressors.  Figure 2(a) demonstrates survival of wild-type YPH250 cells grown with AKG at different concentrations and treated with 10 mM H_2_O_2_. Cell viability in control cultures (without AKG) upon treatment with 10 mM H_2_O_2_ amounted to 45% of the untreated cell viability. The growth with 0.1 mM AKG did not influence yeast susceptibility to 10 mM H_2_O_2_, whereas stress survival of YPH250 cells grown with 1 and 10 mM AKG was 15% and 20% higher, respectively, than that of control cells. Thus, the cultivation in AKG-supplemented medium can enhance resistance of* S. cerevisiae* cells to H_2_O_2_.

Given that the most pronounced effect on yeast resistance to H_2_O_2_ was observed at cultivation with 10 mM AKG, this AKG concentration was used in all next experiments.  Table 2 presents the results of growth in AKG-supplemented medium on yeast survival under followed exposure to different stress factors: 2 mM Fe^2+^, 2 mM Cu^2+^, 20% C_2_H_5_OH, 100 mM menadione, and heat shock (40°C). Survival of* S. cerevisiae* YPH250 cells in control cultures (without AKG) was substantially reduced by treatment with these stressors. Yeast cells grown in the presence of 10 mM AKG showed 22%, 28%, and 45% higher survival after exposure to 20 mM menadione, 2 mM Fe^2+^, and 2 mM Cu^2+^, respectively, as compared to control ones. At the same time, growth on AKG did not affect yeast resistance to 20% ethanol and heat shock.

To find which component of yeast antioxidant defense system could be important for resistance to H_2_O_2_ in AKG-grown wild-type cells, we investigated the strains deficient in certain antioxidant enzymes and Yap1 protein, a transcription factor coordinating yeast response to oxidative stress. The data are presented in Figures 2(b) and 2(c). In control cultures, Δ*sod1*, Δ*cta1*Δ*ctt1*, and Δ*yap1* cells showed higher susceptibility to H_2_O_2_ than parental YPH250 strain. Tolerance to H_2_O_2_ in Δ*sod1*Δ*sod2* and Δ*gsh1* mutants was similar to that of YPH250 strain. Under H_2_O_2_ exposure, Δ*sod1*, Δ*yap1*, and Δ*gsh1* cells grown on AKG showed 1.6–2.6-fold higher stress survival compared to cells in control suspensions. Growth on 10 mM AKG did not affect sensitivity of Δ*sod1*Δ*sod2* and Δ*cta1*Δ*ctt1* cells to H_2_O_2_. The direct addition of 10 mM AKG to incubation medium containing 10 mM H_2_O_2_ enhanced 3.5-fold survival of Δ*cta1*Δ*ctt1* cells from control cultures compared to the same cells subjected only to H_2_O_2_ (Figure 2(c)).

### 3.3. Cultivation with AKG Enhances Antioxidant Defense Capacity in Yeast

**Figure 3:**
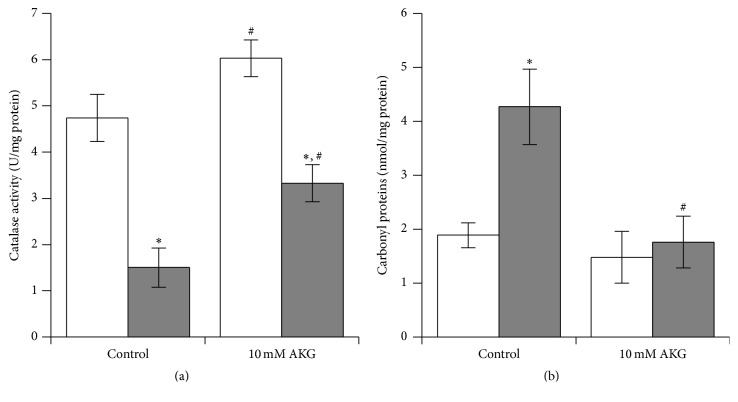
Effect of exposure to 10 mM H_2_O_2_ for 30 min on catalase activity (a) and level of protein carbonyls (b) in* S. cerevisiae* YPH250 cells grown in the absence or presence of 10 mM AKG. Untreated cells (without H_2_O_2_) and cells treated with 10 mM H_2_O_2_ are marked by* white* square and* grey* square bars, respectively. Data are means ± SEM, *n* = 5-6. ^*∗*^Significantly different from respective values of untreated cells and ^#^from respective values of control group (without AKG) with *P* < 0.05 using Student's* t*-test, *n* = 5-6.

**Figure 4:**
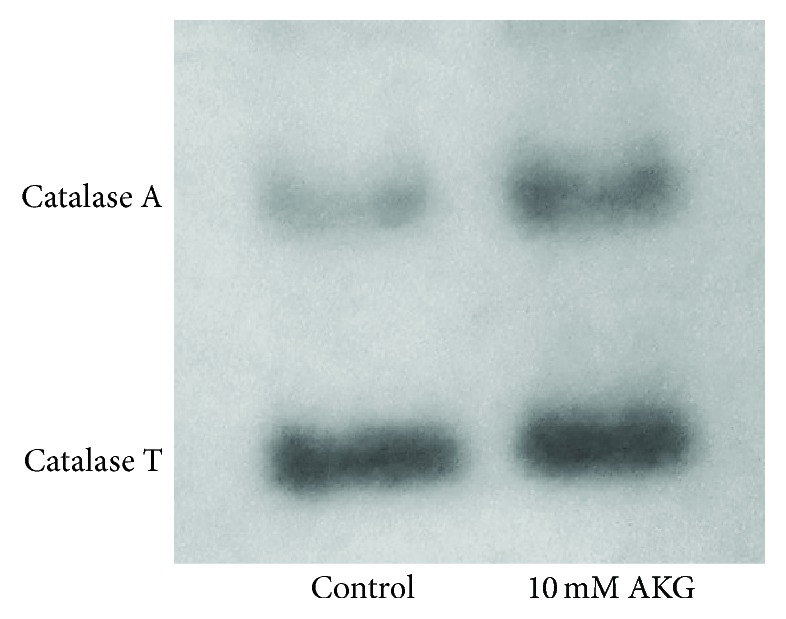
Native PAGE electrophoresis of catalase isoforms in* S. cerevisiae* YPH250 cells grown in the absence or presence of 10 mM AKG. An amount of total protein applied to each well was 10 *µ*g.

To determine if the positive effects of AKG on resistance to stresses were connected with changes in yeast metabolism, in particular with oxidative processes, we measured total metabolic activity of yeast cells, activities of several antioxidant enzymes, and levels of oxidative stress indices. For these experiments, YPH250 cells grown with 10 mM AKG were used. Growth on AKG promoted 30% and 60% higher metabolic activity and concentration of low-molecular mass thiols (L-SH), respectively, compared to control conditions (Table 3). SOD activity was similar, while the activities of glutathione reductase (GR) (Table 3) and catalase (Figure 3(a)) were 3.4-fold and 1.3-fold higher, respectively, in AKG-grown cells compared to control cells. Using native PAGE followed by the quantification of the enzyme activities, we showed that the catalase isoforms' bands were different in cells from control and AKG-supplemented cultures (Figure 4). Peroxisomal catalase A band had 1.4-fold higher intensity in AKG-grown cells, while the cytosolic catalase T band did not differ significantly from the control one. Hence, the higher total catalase activity in AKG-grown cells could be connected with enhanced level of peroxisomal isoform of catalase. Treatment with 10 mM H_2_O_2_ decreased catalase activity in control and AKG-grown cells by 68% and 55%, respectively (Figure 3(a)). The level of protein carbonyl groups (CP) which is widely used for evaluation of the intensity of protein oxidation [21, 22] did not differ in control and AKG-grown cells (Figure 4). Treatment with 10 mM H_2_O_2_ led to a 2.2-fold increase of CP level in control cells and did not change this parameter in cells grown on AKG (Figure 3(b)). The results suggest that AKG-grown cells were more resistant to H_2_O_2_-induced damage than the control ones.

## 4. Discussion

Alpha-ketoglutarate, an endogenous intermediate in the Krebs cycle, is a molecule involved in cellular energetics and amino acid metabolism. Many studies demonstrate that in different cases of induced oxidative stress under in vitro or in vivo conditions, AKG have antioxidant properties [6–11, 17] as well as the capacity of endogenous antioxidant defense thus affecting stress resistance of organisms [12, 13]. In particular, we found previously that AKG-enriched diet enhanced antioxidant defense capacity and cold stress tolerance in young* D. melanogaster* adults [13]. In this work, we showed that growth in AKG-enriched medium increased resistance to stresses in unicellular yeast* S. cerevisiae, *but the effects depended on the concentration of AKG, type of stressors, and gene deficiency in yeast strains. The positive effects of AKG supplement in the medium (1 and 10 mM) on oxidative stress resistance were obtained at relatively high concentrations. It can be connected with the fact that AKG is a charged dicarboxylate molecule and therefore it is not highly membrane permeable. Earlier, exogenous AKG was shown to enter various eukaryotic cells by different active organic anion transporter mechanisms (i.e., different Na^+^ dicarboxylate cotransporter proteins were described) or simply by diffusion at low pH (<3.0) [31]. In* S. cerevisiae*, dicarboxylate transporters have not been studied well and they are mainly represented by low-specific H^+^/dicarboxylate symporters. The transport mechanism is reversible, accumulative, and dependent on the transmembrane gradient of the substrate [32]. Thus, we can suppose that, at higher concentrations in the medium, exogenous AKG can more actively enter the yeast cell leading to reorganization and/or intensification of cellular metabolic processes. Our data on the total metabolic activity support the increase in metabolic rate in yeast cells grown on AKG. The metabolic activity was measured by reduction of colorless tetrazolium salt (ТТС) yielding red formazan. The reaction occurs in the mitochondria where TTC is reduced at different sites along the electron-transport chain [25]. One can suppose that cells grown in the presence of AKG respire more actively than their counterparts grown under control conditions. It is known that the Krebs cycle and respiratory chain are poorly active in* S. cerevisiae* cells growing at high glucose levels. Glucose is preferentially metabolized via glycolysis to form nonfermentable carbon compounds, mainly ethanol. The genes encoding Krebs cycle enzymes are also downregulated by high levels of glucose [33]. In* S. cerevisiae*, the inhibitory effect of glucose is canceled at its concentrations lower than 0.2% and depletion of glucose increases 3–10 times levels of mRNAs of Krebs cycle enzymes [34]. In our experiments, the glucose concentrations at 18 h of growth in the media collected from control and AKG-grown yeast cultures were 0.113 ± 0.028 and 0.104 ± 0.021%, respectively. Hence, the cells were not under glucose repression and characterized by an increase in flux through the Krebs cycle. Therefore, it may be supposed that exogenous AKG can penetrate the cells and be included in Krebs cycle where it is oxidatively decarboxylated to succinyl-CoA by the *α*-ketoglutarate dehydrogenase complex yielding also NADH. In line with this, the ability of AKG to increase respiration and oxidative phosphorylation was found earlier in rat liver [35]. AKG-mediated intensification of mitochondrial respiration can lead to increase in the level of ROS as by-products of respiratory metabolism [18, 25]. In addition, ROS can be formed as side products of AKG oxidative decarboxylation catalyzed by *α*-ketoglutarate dehydrogenase complex [36]. Small increase in ROS level may induce mild oxidative stress which results in the induction of antioxidant defense [1, 13, 18, 19, 37]. This assumption is confirmed by the increased activities of catalase and GR and higher level of low-molecular mass thiols in AKG-grown yeast cells. The low-molecular mass thiols are represented mainly by glutathione and increased level of L-SH could result from either enhanced level of precursors of glutamine or enhanced expression of key enzymes of glutathione biosynthesis [38]. Given that the ability of AKG to serve as a glutamate precursor for glutathione biosynthesis was shown earlier in human erythrocytes [14], we suppose that in our experiments externally added AKG could be used for glutathione biosynthesis resulting in increased L-SH level.

Mild oxidative stress is known to preadapt organisms to lethal oxidative and other stresses [1, 18, 26, 37, 39]. Thus, the increase in antioxidant defense as a result of mild oxidative stress can explain higher resistance of AKG-grown YPH250 cells to H_2_O_2_, menadione, and Fe^2+^ and Cu^2+^ ions. These stressors are directly connected with development of oxidative stress: menadione is a compound generating O_2_^•−^ [39] and Fe^2+^/Cu^2+^ can interact with H_2_O_2_ in Fenton reaction with generation of high reactive hydroxyl radical [1]. Growth with AKG did not improve yeast resistance to ethanol and heat shock. Both ethanol and heat shock can lead to enhanced ROS production, but it seems that other mechanisms of the stressors are more involved in yeast death, and these mechanisms cannot be compensated by the increased antioxidant potential.

It was shown previously that AKG can detoxify H_2_O_2_ and provide an effective protection against oxidative stress [6, 8, 11]. The direct antioxidant action of AKG in our experiments is an unlikely explanation. AKG-grown yeast cells were exposed to stressors after removal of culture medium, and the protective effect could be only ensured by AKG absorbed by the cells during this time. Given that AKG is rapidly metabolized [16, 17], the AKG-grown cells were not able to accumulate it in sufficient amounts.

Exposure to H_2_O_2_ increased CP level in control YPH250 cells but not in AKG-grown cells, which had also better viability under the stress. One may suppose that AKG-treated cells undergo less oxidative damage under H_2_O_2_ exposure. To support this, the substantial decrease in catalase activity in control cells treated with H_2_O_2_ was observed as compared with yeast cells grown in the presence of AKG. The possibility of inactivation of antioxidant enzymes due to oxidative modification has been already shown in our previous studies [29, 40]. Obviously, higher capacity of antioxidant system provides more effective protection in AKG-grown cells against H_2_O_2_-induced damage.

To elucidate which components of antioxidant system were important for realization of stress-protective effects of AKG, we examined resistance to hydrogen peroxide in the yeast strains with defects in antioxidant defense. Growth on AKG increased resistance to H_2_O_2_ in Δ*sod1*, Δ*yap1*, and Δ*gsh1* cells but did not improve stress resistance in Δ*sod1*Δ*sod2* and Δ*cta1*Δ*ctt1* mutants. The results suggest that cytosolic Cu,Zn-SOD, transcriptional regulator Yap1 protein, or *γ*-glutamylcysteine synthetase, an enzyme participating in glutathione synthesis, are not crucial components for the AKG-mediated protective effects. At the same time, the presence of two SOD isoenzymes or catalase isoenzymes is required for stress-protective effects of AKG. The importance of catalase is confirmed by the increased activity of this enzyme, in particular its peroxisomal isoform, in wild-type cells grown on AKG. AKG is metabolized preferentially in mitochondria; therefore the importance of mitochondrial SOD2 in the removal of ROS produced was expected. The importance of both, SOD and catalase, in response of* S. cerevisiae* to H_2_O_2_ is proved by numerous previous studies [19, 39, 40]. Furthermore, since increase in oxidative stress resistance was observed in Δ*yap1* mutant grown on AKG, the induction of antioxidant defense on AKG-supplemented medium seems to occur via not only Yap1-mediated pathways. Other transcriptional factors such as Msn2/4 or Skn7 are known to be involved in adaptive response of yeast to oxidative stress [20, 29]. It should be noted that Δ*cta1*Δ*ctt1* cells treated simultaneously with H_2_O_2_ and AKG survived significantly better compared to cells treated with H_2_O_2_ only. It points out to the antioxidant mode of AKG action in the protection of yeast cells against H_2_O_2_ in cotreatment as it was observed earlier [5]. It also confirms that AKG can act as an antioxidant in combined treatment with oxidants, but its antioxidant properties are not sufficient for protection when cells were grown on AKG, possibly due to fast intracellular metabolism of AKG.

## 5. Conclusions

The obtained results suggest that growth on AKG-supplemented medium increases resistance of yeast* S. cerevisiae* to oxidative stress but not to heat shock and ethanol stress. The protective effect of AKG seems to be due to the enhancement of the cellular energetic metabolism and synthesis of protective proteins. Intensification of metabolic processes is accomplished by the induction of mild oxidative stress resulting in the activation of antioxidant system in yeast cells. In turn, enhanced antioxidant defense confers yeast resistance to oxidants and of transition metal ions. Both isoenzymes of SOD or catalase are required for stress-protective effects of AKG. Given these facts the detailed molecular mechanisms of AKG action, in particular the involvement of master transcription regulators of stress response Msn2/4p and Yap1, should be addressed for future studies.

## Figures and Tables

**Table 1:** Growth of different *S. cerevisiae* strains measured by absorbance at 620 nm (OD_620_) at certain time periods.

Growth conditions	Time, h	Strain					
		YPH250	Δ*cta1*Δ*ctt1*	Δ*sod1*	Δ*sod1*Δ*sod2*	Δ*gsh1*	Δ*yap1*
Control (without AKG)	0	0.095 ± 0.015	0.085 ± 0.010	0.105 ± 0.015	0.087 ± 0.015	0.107 ± 0.015	0.88 ± 0.012
	6	0.117 ± 0.020	0.099 ± 0.011	0.127 ± 0.023	0.097 ± 0.011	0.111 ± 0.021	0.139 ± 0.030
	15	1.012 ± 0.035	0.946 ± 0.099	0.195 ± 0.045^*∗*^	0.658 ± 0.055^*∗*^	1.125 ± 0.065	1.306 ± 0.134
	24	1.178 ± 0.086	1.185 ± 0.058	1.014 ± 0.045	1.119 ± 0.074	1.224 ± 0.125	1.558 ± 1.155
	48	1.575 ± 0.135	1.381 ± 0.125	1.248 ± 0.107^*∗*^	1.321 ± 0.085^*∗*^	1.505 ± 0.110	1.68 ± 0.215

10 mM AKG	0	0.098 ± 0.016	0.083 ± 0.012	0.102 ± 0.017	0.085 ± 0.011	0.101 ± 0.011	0.095 ± 0.013
	6	0.105 ± 0.012	0.098 ± 0.013	0.115 ± 0.018	0.099 ± 0.011	0.115 ± 0.025	0.115 ± 0.025
	15	1.075 ± 0.058	0.954 ± 0.065	0.233 ± 0.048^*∗*^	0.656 ± 0.065^*∗*^	1.154 ± 0.077	1.325 ± 0.145
	24	1.179 ± 0.046	1.195 ± 0.095	1.121 ± 0.098	1.103 ± 0.105	1.248 ± 0.095	1.611 ± 0.173
	48	1.601 ± 0.103	1.377 ± 0.141	1.310 ± 0.138^*∗*^	1.331 ± 0.076^*∗*^	1.550 ± 0.125	1.672 ± 0.185

Cells were cultured in YPD medium supplemented with 10 mM AKG. The starting cell concentration was about 0.3 × 10^6^ cells/mL. Data are means ± SEM, *n* = 3. ^*∗*^Significant difference from respective values for YPH250 (wide type) strain with *P* < 0.05 using Student's *t*-test.

**Table 2:** Effect of different stressors on survival (%) of *S. cerevisiae *YPH250 cells grown in the absence (control) or presence of 10 mM AKG.

Stressor	Control	10 mM AKG
2 mМ FeSO_4_	49 ± 3	63 ± 5^*∗*^
2 mМ CuSO_4_	40 ± 3	58 ± 6^*∗*^
20% ethanol	31 ± 3	28 ± 2
100 mM menadione	54 ± 4	67 ± 4^*∗*^
Heat shock (40°С)	20 ± 3	21 ± 3

Exponentially growing cells were exposed to different stressors for 30 min. Survival of yeast cells in respective suspensions without treatment with stressors was accepted as 100%. Data are means ± SEM, *n* = 5-6. ^*∗*^Significantly different from respective control values with *P* < 0.05 using Student's *t*-test.

**Table 3:** Total metabolic activity and selected characteristics of ROS homeostasis in *S. cerevisiae* YPH250 cells grown in the absence (control) or presence of 10 mM AKG.

Parameter	Control	10 mM AKG
Metabolic activity, OD_485_/10^8^ cells	0.256 ± 0.020	0.327 ± 0.008^**∗**^
SOD, U/mg protein	187 ± 13	185 ± 18
GR, mU/mg protein	25.3 ± 3.9	85.0 ± 17.1^**∗**^
L-SH, nmol/10^8^ cells	20.3 ± 1.8	32.7 ± 1.9^**∗**^

Data are means ± SEM, *n* = 5-6. ^*∗*^Significantly different from respective values in control cultures with *P* < 0.05 using Student's *t*-test.
